# Label-free detection of prostaglandin transporter (SLCO2A1) function and inhibition: insights by wound healing and TRACT assays

**DOI:** 10.3389/fphar.2024.1372109

**Published:** 2024-05-09

**Authors:** Tamara A. M. Mocking, Wieke M. van Oostveen, Jacobus P. D. van Veldhoven, Hugo Minnee, Cynthia M. Fehres, Charles E. Whitehurst, Adriaan P. IJzerman, Laura H. Heitman

**Affiliations:** ^1^ Division of Drug Discovery and Safety, Leiden Academic Centre for Drug Research, Leiden University, Leiden, Netherlands; ^2^ Department of Rheumatology, Leiden University Medical Center, Leiden University, Leiden, Netherlands; ^3^ Immunology and Respiratory Diseases, Boehringer-Ingelheim, Ridgefield, CT, United States; ^4^ Oncode Institute, Leiden, Netherlands

**Keywords:** SLCO2A1, prostaglandin transporter, xCELLigence, PGE2, label-free, prostanoid receptors, wound healing, genetic variants

## Abstract

The prostaglandin transporter (PGT, SLCO2A1) mediates transport of prostanoids (a.o. prostaglandin E2 (PGE_2_)) into cells and thereby promotes their degradation. Overexpression of PGT leads to low extracellular PGE_2_ levels and has been linked to impaired wound healing of diabetic foot ulcers. Inhibition of PGT could thus be beneficial, however, no PGT inhibitors are currently on the market and drug discovery efforts are hampered by lack of high-through screening assays for this transporter. Here we report on a label-free impedance-based assay for PGT that measures transport activity through receptor activation (TRACT) utilizing prostaglandin E2 receptor subtype EP3 and EP4 that are activated by PGE_2_. We found that induction of PGT expression on HEK293-JumpIn-SLCO2A1 cells that also express EP3 and EP4 leads to an over 10-fold reduction in agonistic potency of PGE_2_. PGE_2_ potency could be recovered upon inhibition of PGT-mediated PGE_2_ uptake with PGT inhibitors olmesartan and T26A, the potency of which could be established as well. Moreover, the TRACT assay enabled the assessment of transport function of PGT natural variants. Lastly, HUVEC cells endogenously expressing prostanoid receptors and PGT were exploited to study wound healing properties of PGE_2_ and T26A in real-time using a novel impedance-based scratch-induced wound healing assay. These novel impedance-based assays will advance PGT drug discovery efforts and pave the way for the development of PGT-based therapies.

## 1 Introduction

Prostaglandin E2 (PGE_2_) is an important inflammatory mediator produced by cyclooxygenase 2 (COX2) and mediates various pro- and anti-inflammatory effects such as angiogenesis, cell proliferation, vasodilation, apoptosis and fever (Nakanishi and Rosenberg, 2013). PGE_2_ elicits its actions via activation of four different G protein-coupled receptors, namely, prostaglandin E2 receptors EP1-4, that stimulate distinct G protein signaling pathways. Extracellular levels of PGE_2_ are in part regulated by the prostaglandin transporter (SLCO2A1, PGT) that mediates PGE_2_ uptake into cells and, thereby, promotes its catabolism via oxidation by 15-hydroxyprostaglandin dehydrogenase (15-PGDH) (Nakanishi and Tamai, 2018).

Prostaglandin transporter activity needs to be strictly regulated as both inadequate and increased uptake of PGE_2_ have been associated with a variety of pathologies. Defective PGT due to loss-of function (LOF) mutations elevates extracellular PGE_2_ levels and are consequently linked to inflammatory diseases such as Primary Hypertrophic Osteoarthropathy (PHO) and Chronic Enteropathy Associated with SLCO2A1 (CEAS) (Diggle et al., 2012; Umeno et al., 2015; Umeno et al., 2021). These two diseases have been often mistaken for juvenile rheumatoid arthritis and inflammatory bowel syndrome and are predominantly found in the east-Asian population (Umeno et al., 2015; Nakanishi et al., 2021). In contrast, diabetes patients with diabetic foot ulcers have locally upregulated expression of SLCO2A1 and, consequently, have highly reduced extracellular PGE_2_ levels associated with impaired wound healing due to inability to activate EP4 receptors (Syeda et al., 2012).

Inhibitors for PGT might therefore be a therapeutic strategy to combat diabetic foot ulcers. However, PGT inhibitors are sparsely reported with only a few evident in literature that structurally resemble PGE_2_ (Chi et al., 2006; Chi et al., 2011). Recent screening endeavors of an FDA approved drug library identified some G protein-coupled receptor (GPCR) inhibitors such as cysteinyl leukotriene receptor antagonist pranlukast and angiotensin II type 1 receptor (AT1R) antagonist olmesartan as moderate affinity PGT inhibitors (Kamo et al., 2017). Traditional uptake assays for PGT are troublesome as transported labeled-PGE_2_ is rapidly metabolized and removed from the cell. Screening efforts to identify PGT inhibitors therefore utilize a fluorescent surrogate substrate, 6-carboxyfluorescein (6-CF), which is more stable and has high micromolar affinity for PGT. However, this fluorescent substrate has the disadvantage that uptake is only detectable under acidic conditions (Kamo et al., 2017; Nakamura et al., 2022). Furthermore, it was shown that inhibitory activity from the 6-CF uptake assay did not always correlate well with [^3^H]PGE_2_ uptake assays at physiological pH, suggesting its limited utility as highly informative substrate (Kamo et al., 2017). Likewise challenging are the logistics involved in labelling of prostanoid substrates and using them in uptake assays which together can be costly and labor intensive, plus their inherent lability further hampers the throughput of traditional SLCO2A1 uptake assays. Hence, there is a demand to develop label-free assays to study PGT transport function and enhance throughput.

Recently, application of GPCRs as signaling-sensor for SLC activity has gained increased attention (Dvorak et al., 2021; Sijben et al., 2022b). The so-called ‘transport activity through receptor activation’ (TRACT) assays utilize extracellular levels of substrate rather than intracellular levels to assess transport function. In this case, reduced levels of the extracellular substrate, due to SLC-mediated uptake, will attenuate the GPCR response that is evoked by this substrate. Recently, this method of indirect detection of SLC activity using a label-free impedance-based xCELLigence assay was reported for various SLCs and proven to be a suitable high-throughput alternative for traditional radioligand or fluorescent uptake assays (Vlachodimou et al., 2019; Sijben et al., 2021a; Sijben et al., 2021b; Sijben et al., 2022a; Bongers et al., 2023).

In this study, we exploit the label-free TRACT strategy to measure wild-type and variant prostaglandin transporter activity and inhibition through signaling of prostanoid receptors EP3 and EP4. Three variants were selected, i.e., the PHO mutant L563P, thiazide-induced hyponatremia (TIH) mutant A396T and phosphor-mimic A396E that were recently studied utilizing [^3^H]PGE_2_ uptake assays (Ware et al., 2017; Jimbo et al., 2020). Moreover, we investigated the influence of modulation of extracellular PGE_2_ levels on wound healing using the same impedance-based biosensor to monitor scratch-induced wound healing. This impedance-based wound healing assay provides a real-time and more quantitative detection of scratch closure compared to traditional microscopy-based assays. Both stimulation of prostanoid receptors with PGE_2_ and inhibition of PGT with T26A enhanced proliferation and migration of human umbilical vein endothelial cells (HUVECs) into the scratch area. The established impedance-based TRACT and wound healing assays facilitate label-free detection of PGT activity through prostanoid receptor signaling. These novel assays will hopefully aid drug discovery efforts to identify novel PGT inhibitors.

## 2 Methods

### 2.1 Materials

Jump-In T-Rex HEK293 cells with doxycycline inducible expression of SLCO2A1 (HEK293-JumpIn-SLCO2A1) were generated by and obtained via the Resolute consortium (http://re-solute.eu). Human umbilical vein endothelial cells (HUVEC, CRL-4053), vascular cell basal medium and endothelial cell growth kit - VEGF were obtained from American Type Culture Collection (ATCC, Manassas, VA, USA). Dulbecco’s modified Eagles medium (DMEM), G418 and gelatin were purchased from Merck (Darmstadt, Germany). PGE_2_ was purchased from Selleck chemicals GmbH (Cologne, Germany) and olmesartan was obtained from MedChemExpress (South Brunswick, NJ, USA). cDNA encoding the prostaglandin receptor EP3 (NM_198714.1) or EP4 (NM_000958.3) with N-terminal Myc-tag in pcDNA3.1(+) were purchased from Genscript Biotech B.V. (Leiden, the Netherlands). The PGT inhibitor 4-((4-((2-(2-(2-Benzamidoethoxy)ethoxy)ethyl)amino)-6-((4-hydroxyphenyl)amino)-1,3,5-triazin-2-yl)amino)benzoic acid (T26A) was synthesized in house according to procedures adapted from previously reported literature (For more detail see SI) (Min et al., 2007; Schuster et al., 2015). All other resources were of analytical grade and purchased from standard commercial resources.

### 2.2 Mutagenesis, gateway cloning and cell-line generation

SLCO2A1 mutants A396E, A396T and L563P were generated using QuickChange II site-directed mutagenesis kit (Qiagen) using pDONR221-SLCO2A1 (Addgene, plasmid #131949) as template DNA and primers were obtained from Integrated DNA Technologies (IDT, Leuven, Belgium). Successful mutant generation was confirmed by Sanger sequencing at LGTC (Leiden, the Netherlands). Next, to enable doxycycline-inducible expression of the generated mutants gateway cloning was used to transfer SLCO2A1 mutants from the pDONR vector to a pJTI-R4-DEST-CMV expression vector containing a tet-operon for doxycycline-induction and C-terminal twin-Strep-tag and hemagglutinin (HA)-tag. In brief, 150 ng entry vector pDNOR221-SLCO2A1-mutant (i.e., A396E, A396T or L563P) and 150 ng destination vector pJTI-R4-DEST-CMV were incubated with Gateway LR Clonase II enzyme in TE buffer for 1h at 25°C. The mixture was incubated with Proteinase K solution for 10 min at 37°C prior to transformation into XL-1 blue competent cells. Plasmids were isolated using NucleoBond xtra midiprep kit (Macherey-Nagel, Germany) and sequences were again confirmed by Sanger sequencing. HEK293-JumpIn-parental cells were stably transfected with cDNA of PGT mutants in pJTI-R4 vector using lipofectamine as described previously (Gorostiola González et al., 2023).

### 2.3 Cell culture and transfection

HEK293-JumpIn-SLCO2A1 cells (HEK-JI-SLCO2A1) were cultured in DMEM high glucose supplemented with 10% fetal calf serum (FCS), 2 mM glutamax, penicillin (100 μg/mL) and streptomycin (50 μg/mL) at 37°C with 5% CO_2_. Cells were transiently transfected with 2.5 µg prostaglandin receptor EP3 or EP4 cDNA and 2.5 µg empty pcDNA3.1 vector using the 25 kDa linear PEI method as previously described (Mocking et al., 2019). Cells were cultured for an additional 24 h before use in assays. HUVECs were cultured in vascular cell basal medium supplemented with endothelial cell growth kit–VEGF, 5% FCS, 2 mM glutamax, penicillin (100 μg/mL) and streptomycin (50 μg/mL). For experiments culture medium was replaced with medium containing 2% FCS.

### 2.4 TRACT assay

The Transport activity through receptor activation (TRACT) assays were performed using xCELLigence real-time cell analyzer SP or MP system. The xCELLigence system measures changes in cellular impedance resulting from cell adhesion, cell proliferation or changes in cell morphology. HEK293-JumpIn-SLCO2A1 cells transiently transfected to express prostaglandin EP3 receptor (EP3) or prostaglandin EP4 receptor (EP4) were seeded (70.000 cells/well) in a 96 well E-plate PET in absence or presence of 1 μg/mL doxycycline (dox) to induce PGT expression. After ∼29 h, cells were pretreated with 100 µM olmesartan, 5 µM T26A or vehicle (PBS +0.1% DMSO) for 1 h prior to stimulation of the cells with increasing concentrations of PGE_2_. For inhibition-response curves cells were pretreated with increasing concentrations of inhibitor for 1 h prior to stimulating with 1 nM PGE_2_ (i.e., the concentration that provides best window between -dox and +dox presence). Cellular responses were measured every 30 s for 30 min followed by every minute for 30 min, every 5 min for 1 h and every 15 min up to a total span of 3 h.

### 2.5 Impedance-based scratch-induced wound healing assay

The impedance-based scratch-induced wound healing assay measures the increase in cellular impedance (CI) on a E-wound plate, that only contains electrodes in the scratch area, therefore the CI directly reflects the number of cells that have infiltrated the scratched area. For impedance-based wound healing experiments HUVECs were seeded (40.000 cells/well) onto a 96 well E-wound plate coated with 1% gelatin. The plate was rested for 30 min at room temperature to allow cells to settle before being transferred into the xCELLigence MP cradle in a 37°C 5% CO_2_ incubator. Cell growth and adhesion were monitored every 15 min for a period of 18 h. Next, the E-wound plate was removed from the incubator and homogenous scratches were generated using the AccuWound 96 tool (Agilent, CA, USA). Cell debris was removed by washing the cells with culture medium containing 2% FCS prior to treatment of the cells with 100 nM PGE_2_, 5 μM T26A, 100 nM PGE_2_ and 5 µM T26A or vehicle in medium containing 2% FCS. We selected 100 nM PGE_2_ as this excess concentration was previously shown to successfully induce wound healing for up to 2 days (Syeda et al., 2012; Liu et al., 2015). The plate was returned to the cradle and cellular impedance was monitored every 15 min for a period of 72 h.

### 2.6 Whole cell HA-tag ELISA

An HA-tag enzyme-linked immunosorbent assay (ELISA) was performed to confirm expression of wildtype and mutant PGT variants stably transfected into HEK-JumpIn cells upon induction with 1 μg/mL doxycycline. HEK-JumpIn-SLCO2A1 wildtype, A396E, A396T or L563P were seeded (60.000 cell/well) on a poly-D-lysine coated, transparent, flat-bottom 96 well plate in medium with or without 1 μg/mL dox and grown for an addition 30 h at 37°C 5% CO_2_. Cells were washed with PBS, fixated for 10 min with 4% formaldehyde and permeabilized and blocked using Tris-buffered saline (TBS) with 2% BSA and 0.2% saponin for 1 h. Next, cells were incubated with 1:2500 primary Rabbit anti-HA polyclonal antibody (Invitrogen, Carlsbad, CA, United States) in assay buffer (TBS with 0.1% BSA) for 1 h at RT. Cells were washed with assay buffer prior to incubation with 1:6000 Goat-anti-rabbit HRP-conjugate antibody (Brunschwig Chemie, Amsterdam, Netherlands) in assay buffer for 30 min at RT. Immunoreactivity was detected by incubating with 3,3′,5,5′-tetramethylbenzidine (TMB) for 2 min before quenching with equal volume of 1M H_3_PO_4_. Absorbance was measure at 450 nm using a Wallac EnVision multimode plate reader (PerkinElmer, Groningen, the Netherlands).

### 2.7 Data analysis and statistics

All data was analyzed using Graphpad prism v9.0 (Graphpad software Inc, San Diego, CA, USA). xCELLigence data was recorded and normalized to the timepoint prior to stimulation or scratch to obtain normalized cell index (nCI) or delta CI (ΔCI) using RTCA v2.0 (SP) or RTCA software Pro V2.8.0 (MP) for TRACT and wound healing assays, respectively. TRACT-data was corrected for any ligand-independent effects by subtraction of the vehicle-induced response. From the vehicle-corrected nCI data the net area under the curve (AUC) over the first 3 h was calculated to generate concentration-response curves. To obtain potency (pEC_50_ or pIC_50_) values, concentration-response data was fitted to a non-linear regression three parameter response model. pIC_50_ stands for −log(IC_50_) where IC_50_ is the concentration (in mol/L) required to inhibit the maximal response by 50%. For impedance-based wound healing assays data was analyzed by calculating either the AUC from scratch up to 12, 24 or 72 h after scratch or by taking the ΔCI at timepoint 24 h after scratch and calculating the % wound healing, where maximal ΔCI at 72h was set at 100%. Statistical analyses were performed using Graphpad Prism v9.0 and specified in the legend of each figure.

## 3 Results

### 3.1 Validation of label-free TRACT assay for the prostaglandin transporter SLCO2A1

**FIGURE 1:**
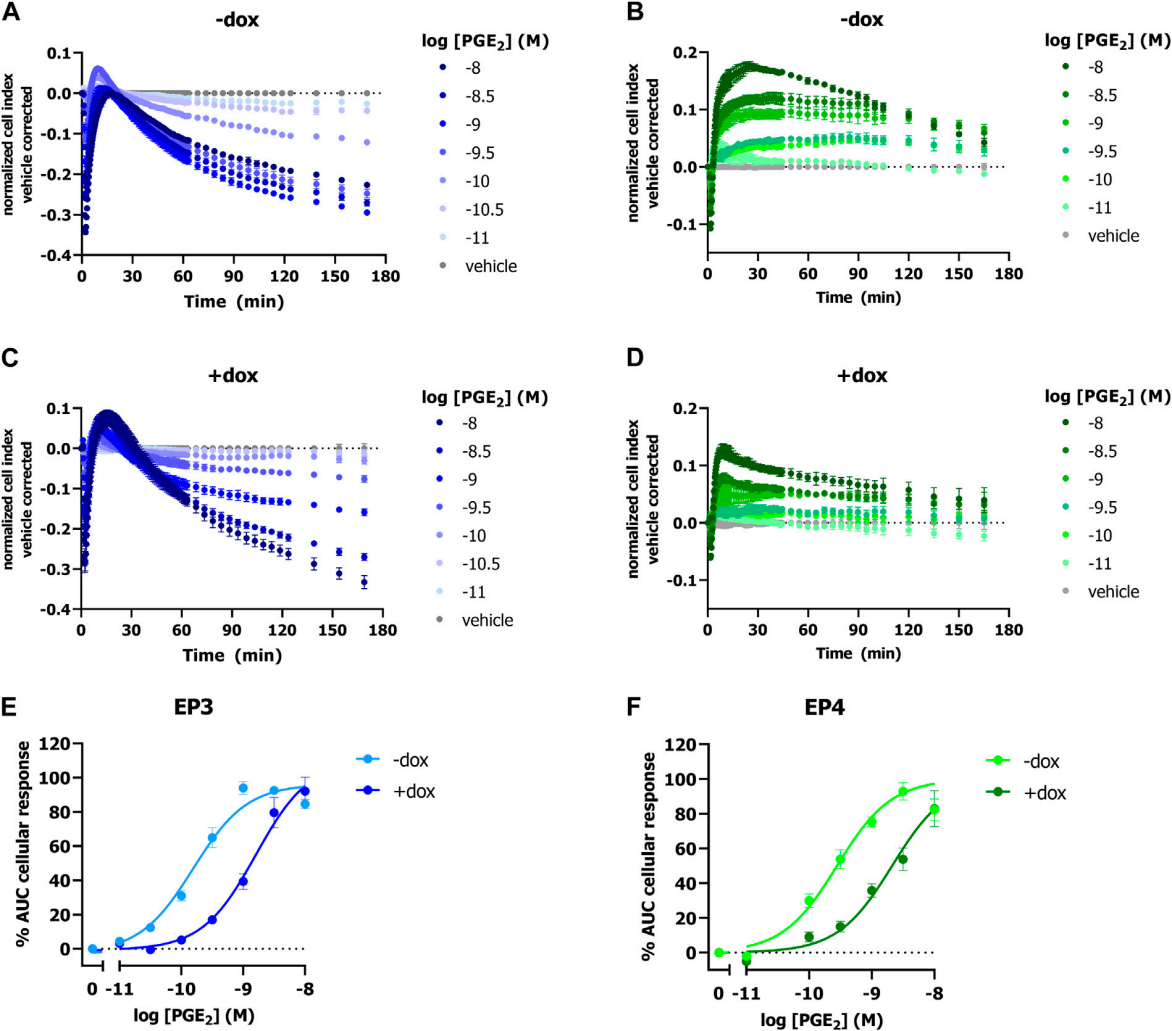
Response of PGE_2_ on EP3 or EP4 expressing HEK-JumpIn-SLCO2A1 cells in absence (-dox) and presence (+dox) of doxycycline-induced PGT expression as measured by xCELLigence. Representative time traces of PGE_2_-mediated response in absence **(A,B)** or presence **(C,D)** of dox-induced SLCO2A1 expression in HEK-JI-SLCO2A1 cells with transient EP3 **(A,C)** or EP4 receptor **(B,D)** expression. Concentration-response curves of PGE_2_ on transient EP3 **(E)** or EP4 **(F)** expressing HEK-JumpIn-SLCO2A1 cells. Data shown **(E,F)** are mean ± S.E.M. of seven experiments performed in duplicate.

To investigate the potential of a label-free impedance-based TRACT assay for PGT using endogenously expressed prostanoid receptors, we selected HEK293-JumpIn-SLCO2A1 cells with dox-inducible expression of the prostaglandin transporter and endogenous expression of prostaglandin E2 receptor EP2 (EP2). Cells were seeded onto a 96 well E-plate and the next day cells were stimulated with increasing concentrations of PGE_2_. PGE_2_ addition to non-induced cells resulted in a clear dose-dependent cellular response in the impedance-based assay (Supplementary Figure S1A). The endogenously expressed EP2 receptors did not allow to detect a sufficient decrease in PGE_2_ levels upon induction of PGT, as no clear rightward shift of the PGE_2_ concentration-response was observed (Supplementary Figure S1). Therefore, HEK-JumpIn-SLCO2A1 cells were transiently transfected to either express the high affinity prostaglandin receptor EP3 or EP4 as these receptors could act as more sensitive sensors to detect changes in extracellular PGE_2_ levels. Interestingly, the obtained cellular impedance traces upon treatment with PGE_2_ were different for activation of G_i_-coupled EP3 receptor and G_s_-coupled EP4 receptor. For EP3, addition of PGE_2_ induced an initial drop in nCI followed by a recovery to baseline within the first 10 min, which was then followed by a gradual decrease in vehicle-corrected nCI up to 180 min (Figure 1A). In contrast, EP4 activation by PGE_2_ mediated a steep increase in vehicle-corrected nCI reaching plateau after approximately 30 min followed by a gradual steady decrease in nCI back to baseline up to 180 min (Figure 1B). Thus, overexpression of EP3 and EP4 in HEK-JI-SLCO2A1 cells resulted in a clear dose-dependent decrease or increase in cellular response for EP3 and EP4, respectively, as measured by xCELLigence (Figures 1A, B). Next, the effect of PGT expression on these cells was investigated by dox-induction, which reduced the concentration-dependent cellular PGE_2_ response (Figures 1C, D). Both EP3 and EP4 expressing cells displayed an over 10-fold reduced potency for PGE_2_, as demonstrated by a rightward shift of the PGE_2_ concentration-response curve upon induction of PGT (Table 1; Figures 1E, F). Taken together, PGT-mediated PGE_2_ uptake can be detected as attenuation of the GPCR response using either EP3 or EP4 receptors as PGE_2_ sensor in the impedance-based assay.

**TABLE 1:** Potency of PGE_2_ on prostanoid receptor EP3 or EP4 in absence and presence of dox-induced PGT expression as measured by impedance-based TRACT assay. Data are shown as mean ± SEM of (n) experiments performed in duplicate.

	EP3	EP4
	pEC_50_ ± SEM (n)	
-dox	9.8 ± 0.1 (7)	9.6 ± 0.1 (7)
+dox	8.8 ± 0.1 (7)***	8.5 ± 0.2 (7)***
+dox +100 µM olmesartan	9.5 ± 0.1 (6)^‡‡‡^	9.1 ± 0.2 (4)
+dox + 5 µM T26A	9.2 ± 0.2 (3)*	9.0 ± 0.1 (3)

One-way ANOVA, with Dunnett’s post-hoc test, ***p < 0.001, *p < 0.05 (compared to -dox) and ^‡‡‡^ *p* < 0.001(compared to + dox).

**FIGURE 2:**
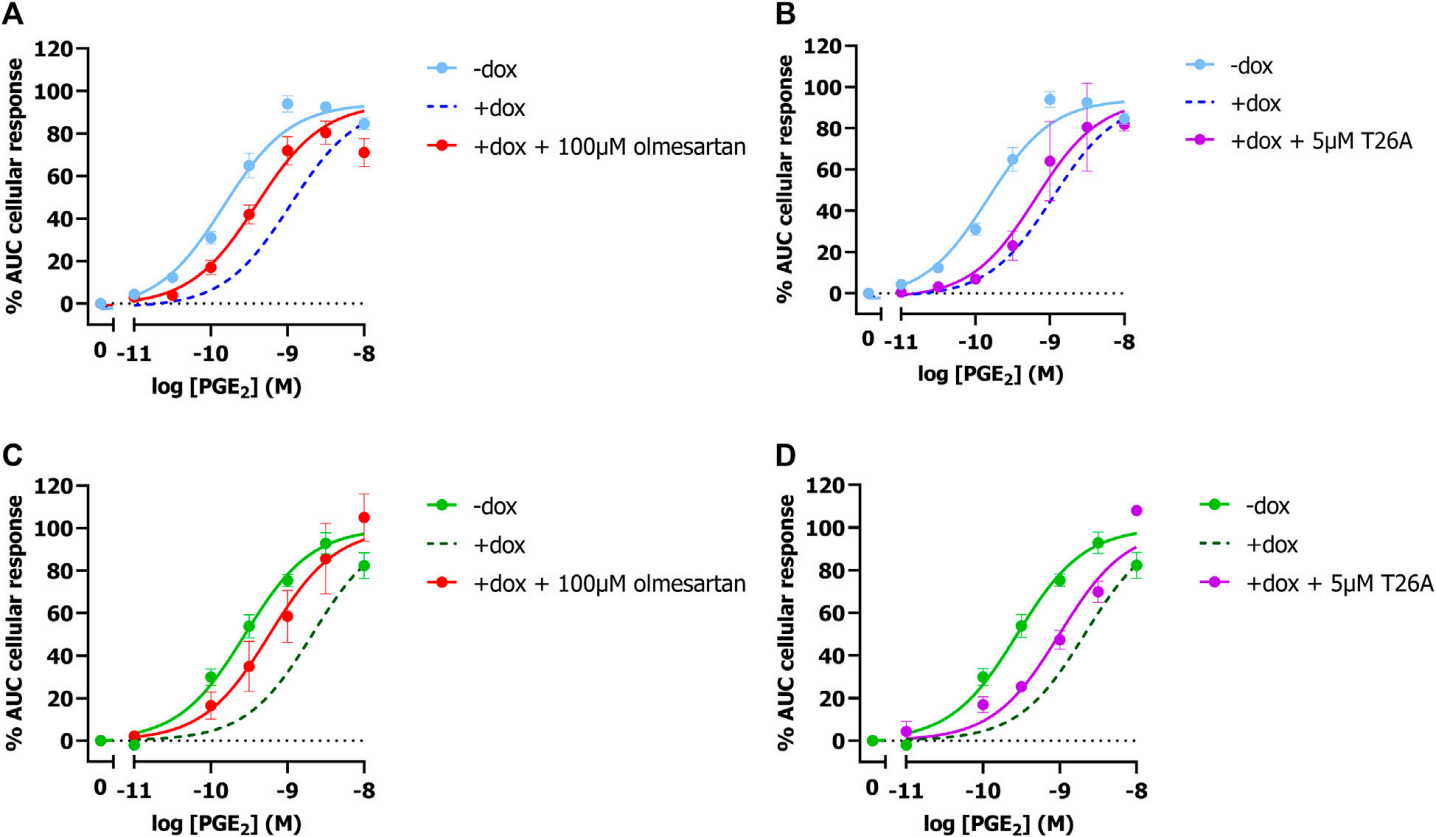
Concentration-response curves of PGE_2_ in absence and presence of PGT inhibitors Olmesartan **(A,C)** and T26A **(B,D)** on induced and non-induced HEK-JumpIn-SLCO2A1 cells. Concentration response-curves of PGE_2_ on EP3 **(A,B)** or EP4 **(C,D)** expressing cells. Dotted line represents the dox-induced PGE_2_ response in absence of inhibitors (Figures 1E, F). Data shown are mean ± S.E.M. of at least three experiments performed in duplicate.

To validate that the observed potency shift could be attributed to PGT-mediated PGE_2_ uptake in these cells, dox-induced HEK-JumpIn-SLCO2A1 cells expressing either EP3 or EP4 were pretreated with PGT inhibitor olmesartan prior to stimulation with increasing concentration of PGE_2_. Indeed, upon pretreatment with 100 µM olmesartan a leftward shift of the PGE_2_ concentration-response curve was observed with a 5.2-fold and 4.2-fold shift in potency for EP3 and EP4, respectively (Figures 2A, C; Table 1). The angiotensin II receptor type 1 (AT1R), the high-affinity target of olmesartan, is not expressed on HEK-JumpIn cells according to transcriptomics data (BioSamples database: SAMN11893676, SAMN11893683 and SAMN11893690). In agreement, pretreatment of either induced or non-induced HEK-JumpIn-SLCO2A1 cells with increasing concentrations of olmesartan did not induce changes in cellular impedance (Supplementary Figure S2). In addition, dox-induced cells pretreated with 5 µM of selective PGT inhibitor T26A displayed a modest 2.5-fold and 3.2-fold increased potency for EP3 and EP4, respectively (Figures 2B, D). The ability of olmesartan and T26A to partially revert the PGE_2_ response back towards a non-induced response (i.e., absence of PGT), corroborates the establishment of a TRACT assay for PGT.

**FIGURE 3:**
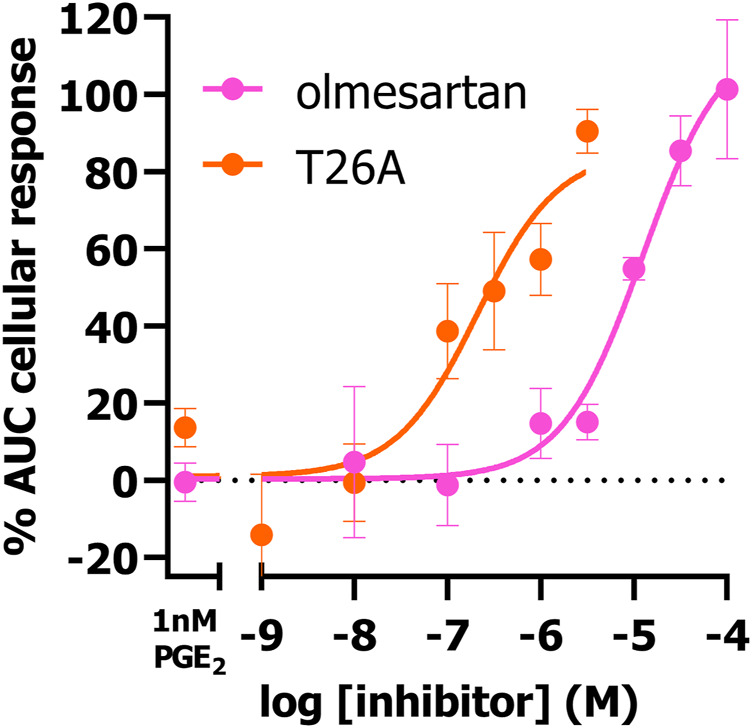
Concentration-inhibition curves of olmesartan and PGT inhibitor T26A on dox-induced HEK-JumpIn-SLCO2A1 cells transiently expressing EP4 receptor as measured using xCELLigence-based TRACT assay. Data shown are mean ± S.E.M. of at least three experiments performed in duplicate.

Next, we investigated if the presented TRACT assay could be used to determine the potency of PGT inhibitors utilizing the EP4 receptor as signaling partner. Of interest, EP4 was selected over EP3 to determine inhibitory potencies as the observed response was more robust and due to its link to impaired wound healing (*vide infra*). We selected 1 nM PGE_2_ as preferred concentration to stimulate EP4 expressing cells in inhibition experiments as this concentration resulted in the largest shift (window) in PGE_2_-mediated AUC cellular response between uninduced (-dox) and PGT expressing cells (+dox) (Figure 1F). HEK-JumpIn-SLCO2A1 cells transiently expressing EP4 receptor were pretreated with increasing concentrations of PGT inhibitor olmesartan or T26A for 1 h prior to stimulating the cells with 1 nM PGE_2_ (Figure 3). Both inhibitors displayed dose-dependent inhibition of PGT, observed as an increase in cellular response, with pIC_50_ = 5.0 ± 0.0 for olmesartan and pIC_50_ = 6.7 ± 0.2 for T26A, potencies that are comparable to values of traditional [^3^H]PGE_2_ uptake assays (Chi et al., 2011; Kamo et al., 2017). This shows that the impedance-based TRACT assay is a useful (orthogonal) assay to determine the potency of PGT inhibitors.

### 3.2 PGT-based TRACT assay enables study of functional activity of natural variants and mutants

**FIGURE 4:**
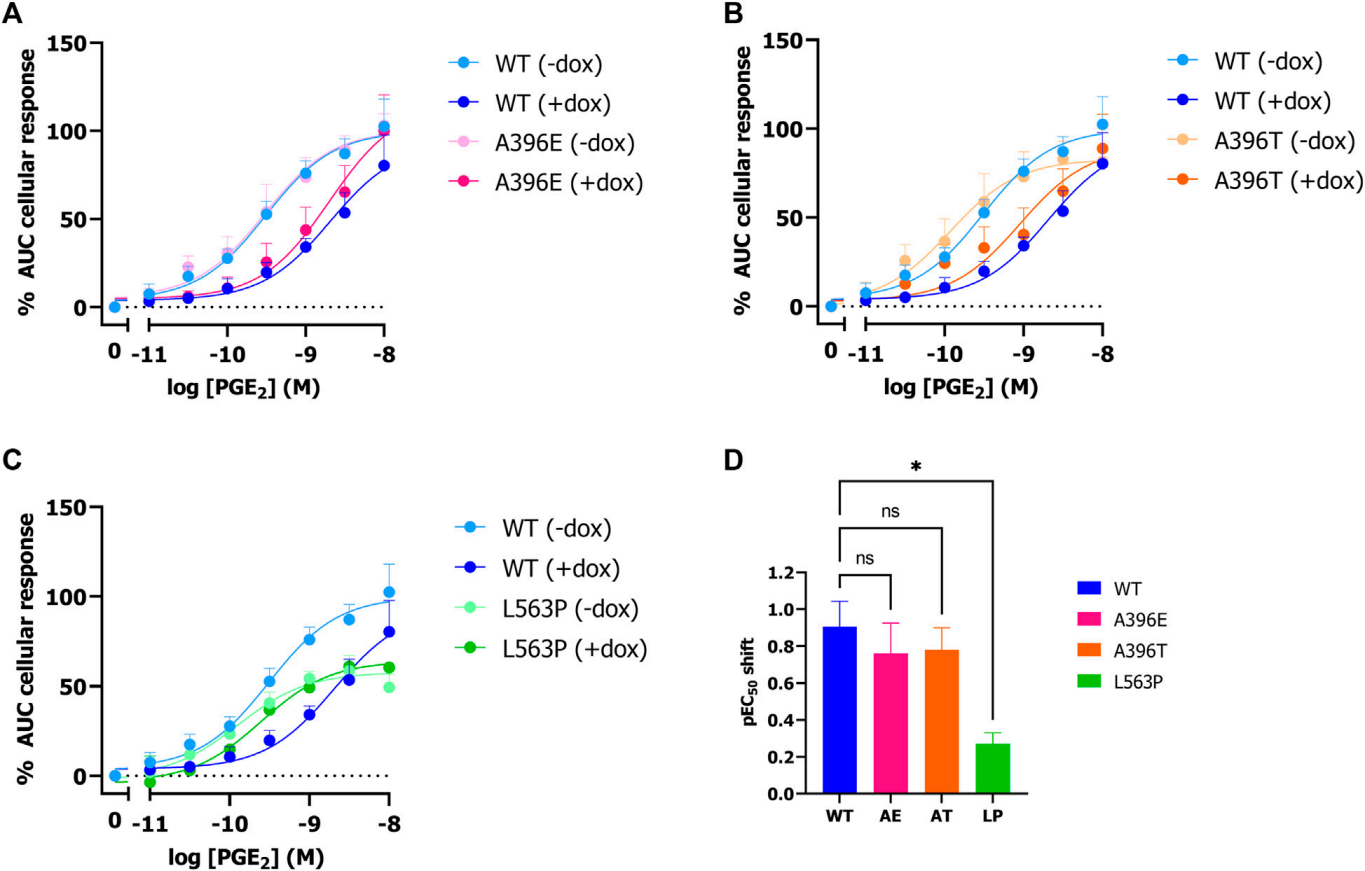
Effect of SNPs A396E, A396T and L563P on PGT activity as measured by impedance-based TRACT assay. Response of PGE_2_ on wildtype and mutant PGT in the absence and presence of doxycycline on EP4 expressing HEK-JumpIn cells **(A–C)**. Potency shift of PGE_2_ upon induction of SLCO2A1 WT or mutants **(D)**. Data shown are mean ± S.E.M. of at least three experiments performed in duplicate. One-way ANOVA with Dunnett’s post-hoc test *p < 0.05.

Next, it was assessed whether natural and disease-related PGT variant activity can be studied using the established label-free impedance-based TRACT assay. To this end, HEK-JumpIn-SLCO2A1 cells were generated that expressed either PHO mutant L563P, thiazide-induced hyponatremia (TIH) mutant A396T or A396E, i.e., a phospho-mimic of A396T that was reported to have decreased uptake activity). Stable transfection of each of the three PGT variants into HEK-JumpIn cells resulted in similar expression levels to wildtype PGT upon dox-induction as confirmed by HA-tag ELISA (Supplementary Figure S3). Both TIH-mutant A396T and its phosphomimic A396E display a 6-fold (pIC_50_ shift 0.8) reduced potency of PGE_2_ upon dox-induced PGT expression indicating that they were still capable of transporting PGE_2_ (Figures 4A, B, D). Moreover, the observed potency shift for these mutants was not significantly different from wildtype. In contrast, the loss of function mutant L563P abrogated the shift in PGE_2_ potency upon dox-induction, consistent with loss of PGE_2_ transport, and this loss was not related to less L563P expression as confirmed by whole cell ELISA (Figure 4C; Supplementary Figure S3). Together, these data confirm that the TRACT assay can be utilized to assess uptake activity of PGT disease-related and natural variants.

### 3.3 PGE_2_ and T26A increase wound healing as measured by xCELLigence

**FIGURE 5:**
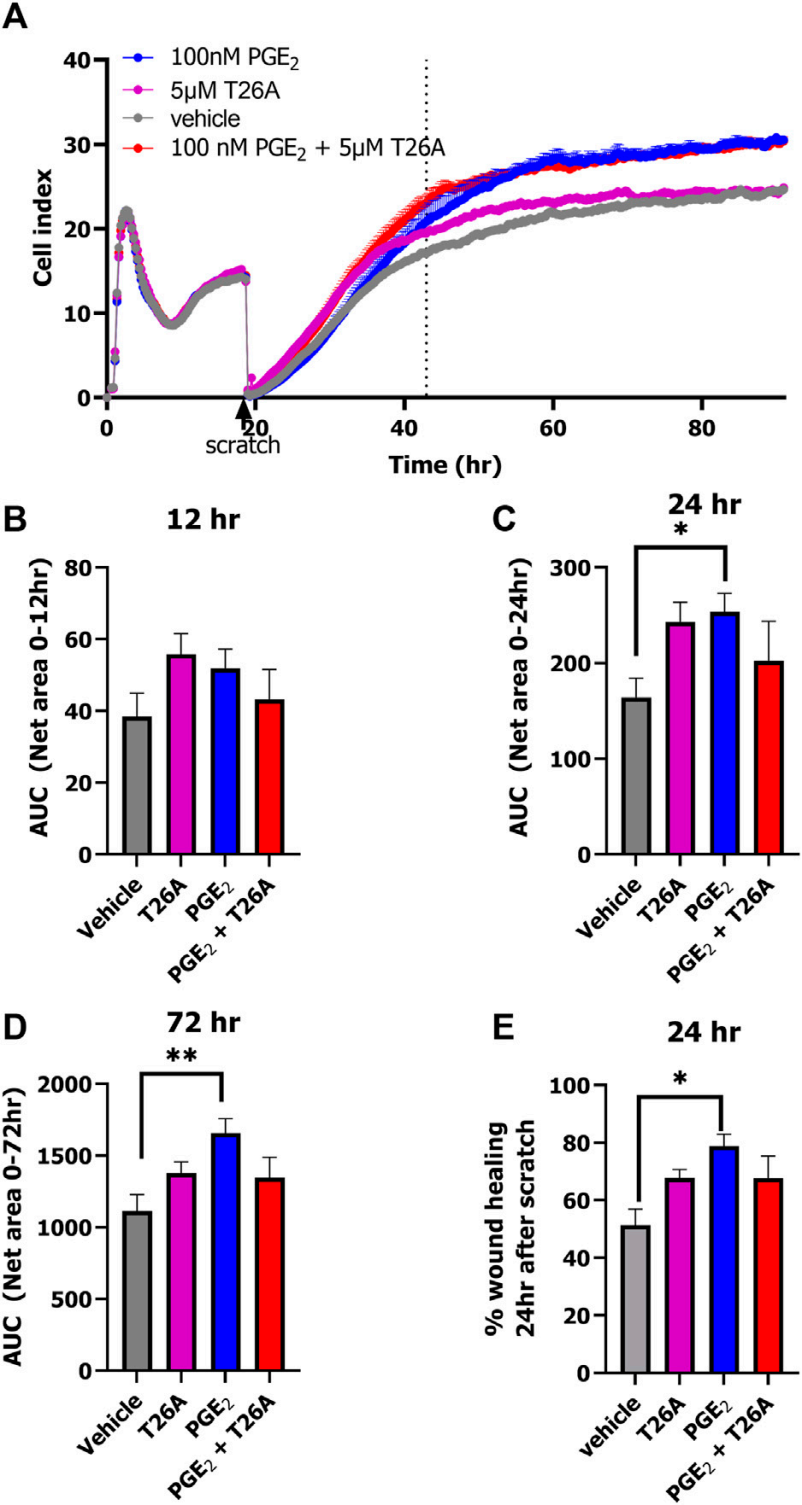
xCELLigence-based scratch-induced wound healing of HUVECs endogenously expressing PGT and prostanoid receptors. Representative time trace of HUVEC seeding, proliferation followed by scratch-generation and stimulation of cells with 100 nM PGE_2_, 5µM T26A, 100 nM PGE_2_ and 5 µM T26A or vehicle-induced scratch healing as measure by xCELLigence **(A)**. Dotted line indicates the timepoint 24 h after scratching the cells. Area under the curve (AUC) analysis of scratch closure over 12 h **(B)**, 24 h **(C)**, or 72 h **(D)**. Wound healing (%) progress at timepoint 24 h after scratch **(E)**. Data shown are mean ± SEM of at least three experiments performed in triplicate. One-way ANOVA with Dunnett’s post-hoc test *p < 0.05, **p < 0.01.

Lastly, we explored the opportunity to detect wound healing in real-time with the impedance-based technology using HUVECs endogenously expressing prostanoid receptors and PGT. HUVECs were seeded onto an E-wound plate and grown for 18 h until a plateau was reached (Figure 5A; Supplementary Figure S4). Identical scratches were induced by the AccuWound 96 tool and scratch closure was followed in real-time for 3 days in the presence of 100 nM PGE_2_, 5 μM T26A, both 100 nM PGE_2_ and 5 µM T26A or vehicle (Figure 5A; Supplementary Figure S4). As HUVECs also express the Angiotensin II type 1 receptor we omitted olmesartan in this wound healing assay to avoid non-PGE_2_ mediated effects. Treatment with either PGE_2_, T26A or both accelerated invasion of the lesion area by cells and thus scratch closure within the first 24 h after scratch generation as compared to vehicle treated cells (Figures 5A–C). However, calculating the AUC after 24 and 72 h (i.e., total measured time) only PGE_2_ addition mediated a significantly increased scratch closure as the effect of T26A seemed to diminish after 48 h (one-way ANOVA with Dunnett’s post-hoc test) (Figures 5A, D). However, when comparing the % wound healing at 24h after scratch generation T26A and co-addition of PGE_2_ and T26A presented a clear trend of increased scratch closure compared to vehicle treated cells (Figure 5E). Interestingly, PGE_2_ accelerated the scratch repair, as a fully healed scratch apparent from the curve plateau was evident at approximately 48 h, while vehicle-treated cells did not reach plateau until after 72 h (Figure 5A). Taken together, enhanced activation of prostanoid receptors by either addition of extracellular PGE_2_ or reduced uptake and breakdown due to PGT inhibition with T26A accelerated wound healing.

## 4 Discussion

Prostanoid uptake facilitated by PGT (SLCO2A1) is a pivotal process to control inflammation, and severe dysregulation of this process leads to disease pathologies (Seifert et al., 2012; Nakanishi and Tamai, 2018; Yanai et al., 2019; Umeno et al., 2021). The reduced healing capacity of diabetic foot ulcers was recently associated with local upregulation of PGT expression and compromised the ability of PGE_2_ to stimulate wound healing effects by activation of prostanoid receptors (Syeda et al., 2012; Theocharidis et al., 2020; Nakanishi et al., 2021). Therapeutic intervention to promote the healing process of these foot ulcers could be mediated by topically inhibiting PGT to normalize extracellular PGE_2_ levels. Unfortunately, lack of suitable high affinity inhibitors for PGT and disadvantages of conventional uptake assays have hindered the discovery of novel PGT inhibitors. Hence, there is a need for novel higher throughput assays that facilitate study of PGT activity over time without the need to label the substrate. Recently, we developed label-free impedance-based TRACT assays for monoamine transporters SLC6A2 and SLC6A3 and equilibrative nucleoside transporter 1 (ENT1, SLC29A1) that facilitate SLC transport function to be detected via substrate-mediated GPCR activation (Vlachodimou et al., 2019; Sijben et al., 2021a; Sijben et al., 2021b). Here, we established a label-free impedance-based TRACT assay for the prostaglandin transporter (SLCO2A1) that enables detection of its uptake activity and inhibition. Moreover, we further exploited the impedance-based technology in an endothelial cell-based scratch-induced wound healing assay for PGT.

The TRACT assay utilizes prostanoid receptors as sensors for extracellular levels of substrate rather than intracellular levels to assess transport function, circumventing the rapid metabolism and disposition of PGE_2_ from the cells. The high affinity prostanoid receptors EP3 and EP4 were suitable sensors for PGT-mediated PGE_2_ uptake (Figure 1). The reversely oriented cellular impedance responses upon activation of these receptors can be explained by the fact that EP3 receptors are known to result in G_i_-activation, while EP4 receptors predominantly activate G_s_ proteins, that are known to cause cytoskeletal rearrangements leading to an increased and decreased cellular response in the xCELLigence system, respectively (Nishigaki et al., 1996; Tsuboi et al., 2002; Scott and Peters, 2010). Interestingly, this differential signaling did not influence the observed potency shift as both receptors display an approximate 10-fold decrease in PGE_2_-potency upon PGT overexpression (Table 1). Advantageously, their high affinity for PGE_2_ as compared to other EP receptors (Abramovitz et al., 2000) enabled them to sense changes in extracellular PGE_2_ levels already in the sub- to low-nanomolar range (Figure 1). Thus, high affinity EP receptors are needed to accurately detect PGT-mediated transport in the TRACT assay.

Next, it was established by using PGT inhibitors that the observed reduction in PGE_2_ potency was indeed due to PGT-mediated transport. While the non-selective PGT inhibitor olmesartan almost fully shifted the PGE_2_ concentration-response curve back to non-induced condition, selective PGT inhibitor T26A displayed only a marginal 2.5–3.2-fold leftward-shift (Figure 2). A previously published assay measuring EP1-mediated Ca^2+^ release to detect PGT activity similarly displayed only a partial leftward shift (1.7-fold) upon pretreatment with 5 µM T26A (Chi et al., 2014). In our hands this concentration caused solubility issues, while the compound did display full inhibition of PGT activity in competition with a low (1 nM) PGE_2_ concentration (Figure 3). The TRACT assay indirectly measures PGT activity and, consequently is therefore reliant on the expression levels of EP3 or EP4 receptors, given that GPCR signaling responses are known to be dependent on receptor expression levels. Even though EP4 is known to rapidly desensitize upon activation it has been reported to have a large receptor reserve (Wilson et al., 2004; Chi et al., 2014). Therefore, our TRACT assay was not hampered by receptor desensitization, as the observed shift in PGE_2_ potency for EP4 signaling was still detectable after 3 h (Figures 1B, D, F). Moreover, potencies of tested PGT inhibitors were comparable to those observed in conventional radioligand uptake assays using radiolabeled PGE_2_ (Chi et al., 2011; Kamo et al., 2017). Suramin, another PGT inhibitor discovered by Kamo et al. was not suitable for analysis in the TRACT assay due to its promiscuous nature of interacting with G proteins and other off-target proteins such as P2Y receptors that are endogenously expressed on our HEK-JumpIn cells (Beindl et al., 1996; Freissmuth et al., 1996; Kamo et al., 2017; Sriram and Insel, 2018; Wiedemar et al., 2020). Therefore, the established TRACT assay for PGT mediates a robust response and might be used to study PGT inhibitors, also on cells endogenously expressing high affinity EP receptors.

The utility of the TRACT assay was also explored to assess the activity of three previously characterized PGT mutants, and it yielded similar results as in regular uptake assays. Indeed, loss-of-function mutation L563P did not result in significant uptake of PGE_2_ (Jimbo et al., 2020). However a slight though not significant decrease in E_max_ was observed for both - dox and +dox conditions for this mutant compared to wild-type SLCO2A1 (Figure 4C). In contrast, TIH mutants A396T and A396E displayed PGT-mediated PGE_2_ transport function that was slightly reduced though not significantly different from wildtype (Figure 4). In contrast to Ware and others, who reported decreased uptake activity (i.e., transport rate) via the A396E mutant in a radiolabeled PGE_2_ uptake assay (Ware et al., 2017), we did not observe a significant difference between wildtype, the A396T mutant and its phosphomimic A396E in our TRACT assay, which might be due to different expression levels.

Alterations in PGE_2_ levels due to PGT-mediated uptake and degradation highly influence physiological processes such as inflammation, blood pressure, vasodilatation and angiogenesis (Chi et al., 2015; Trau et al., 2016). Interestingly, in the complex process of wound healing PGE_2_ is promoting both pro- and anti-inflammatory effects by activation of EP3 and EP4 receptors. Both receptors are known to induce cell migration and insufficient activation of these receptors impairs the healing process as was observed in diabetics (Syeda et al., 2012; Chi et al., 2014). It has been shown that inhibition of PGT expression and direct inhibition with PGT blockers boost cell proliferation and re-epithelialization, and enhance vascularization and blood flow thereby promoting wound healing (Syeda et al., 2012; Liu et al., 2015; Lu et al., 2021). Here, in a simplified scratch-induced wound healing model, we demonstrated that scratch closure can be followed in real-time with the novel impedance-based, scratch-induced wound healing assay utilizing HUVECs endogenously expressing PGT and prostanoid receptors (Trau et al., 2016; Liang et al., 2021). Scratch-induced wound healing assays are commonly endpoint assays that are evaluated by microscopy after 12–24h, with the percentage of scratch closure being estimated through image-based analysis. The impedance-based wound healing assay provides a more accurate determination of scratch closure as attachment of the cells in the scratch area will impede the electron flow over the gold-plated wells, resulting in changes in cellular impedance. Thereby this method provides a more quantitative method to evaluate wound closure, which is followed in real time. Hezinger et al. (2023) provided evidence that impedance-based scratch assays yield similar results as the traditional microscopy-based wound healing assay as observed for NOD1 silencing in HeLa cells (Hezinger et al., 2023). Wound healing occurs more rapidly when extracellular PGE_2_ levels are increased either due to direct addition of 100 nM PGE_2_ or by a decrease in PGT mediated uptake and breakdown of PGE_2_ due to T26A mediated inhibition of PGT (Figure 5). However, T26A only advanced wound healing within the first 24 h, while PGE_2_ significantly increased wound healing for the full 72 h compared to vehicle. Combined addition of PGE_2_ with the PGT inhibitor T26A reached similar levels as PGE_2_ alone but could not further increase wound healing. This can be explained by the fact that 100 nM PGE_2_ is already an excess concentration of PGE_2_ and therefore already is expected to lead to full activation of prostanoid receptors. Indeed, these results for PGT involvement in wound healing are comparable to data from previously reported wound healing assays in cells endogenously expressing PGT and prostanoid receptors (Syeda et al., 2012; Liu et al., 2015; Nakanishi et al., 2017).

In conclusion, the impedance-based, label-free technology provides an innovative approach to study PGT activity in both TRACT and wound healing format, eliminating the need for labelled substrates. The impedance-based wound healing assay described in the current study provides a more quantitative method to determine scratch closure than conventional microscopy-based wound healing assays. Moreover, the impedance-based assays facilitate real-time detection of PGT activity of wildtype protein and its natural and disease-related variants. Finally, the established TRACT and wound healing assays provide useful formats for the detection of novel PGT inhibitors as future drug candidates.

## Data Availability

The original contributions presented in the study are included in the article/Supplementary Material, further inquiries can be directed to the corresponding author.
