# Supplementary material for: Label-free detection of prostaglandin transporter (SLCO2A1) function and inhibition: insights by wound healing and TRACT assays
Source: Front Pharmacol. 2024 May 9;15:1372109. doi: 10.3389/fphar.2024.1372109 (PMC11111933; doi:10.3389/fphar.2024.1372109)
Supplement: Supplementary file 1 [file DataSheet1.PDF]

Supplementary Material

1 Supplementary figures

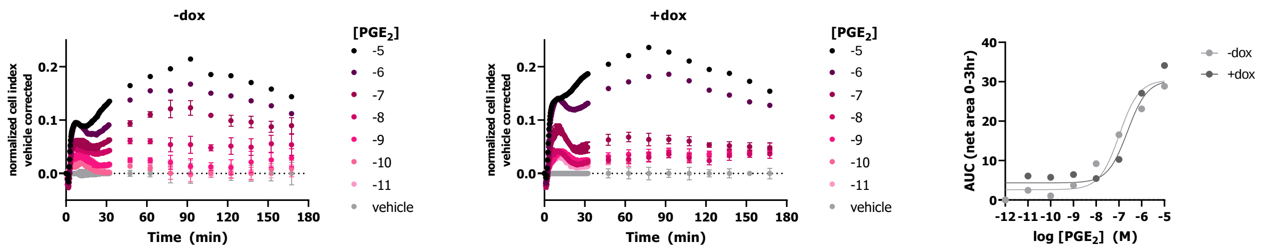

Sfigure 1: PGE_2 response on HEK-JumpIn-SLCO2A1 cells as measured by xCELLigence. Representative time traces and concentration-response curve of PGE_2-mediated effect in absence and presence of dox-induced SLCO2A1 expression. Data shown are means of a representative experiment performed in duplicate.

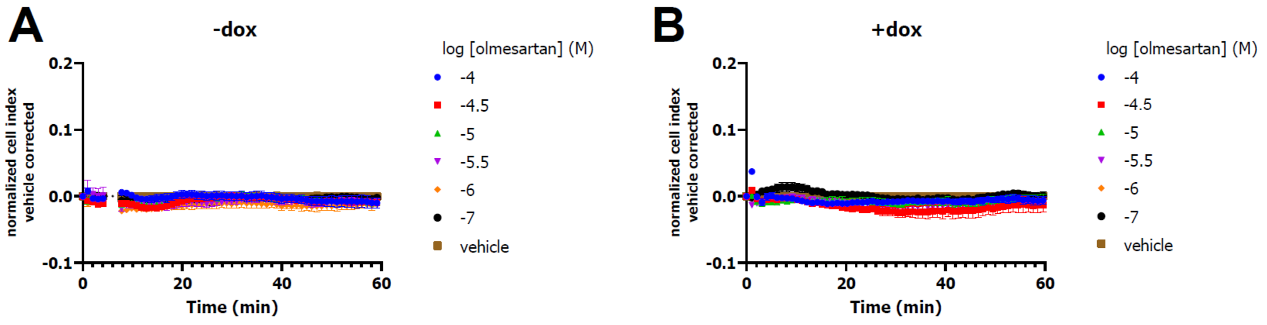

Sfigure 2: Representative time traces of Olmesartan response in HEK-JumpIn-SLCO2A1 cells transiently expressing EP4 receptor in absence (A) and presence (B) of doxycycline-induced SLCO2A1 expression. Cells were treated with increasing concentrations of Olmesartan.

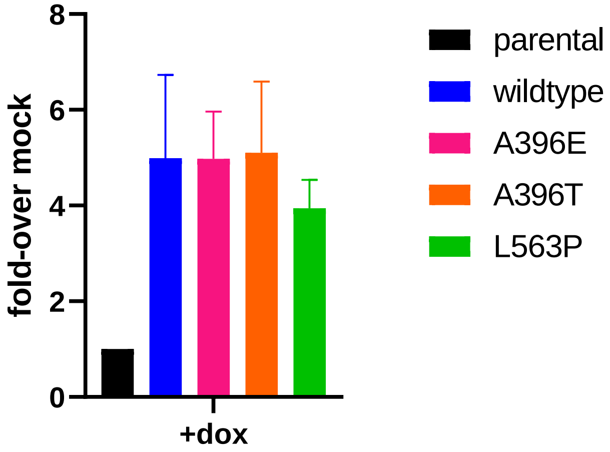

Sfigure 3: Whole-cell HA-tag ELISA of HEK-JumpIn-parental cells, and those stably transfected with wildtype or one of three SLCO2A1 variants, i.e.A396E, A396T or L563P. Data shown are mean ± SD of two experiments performed in quintuplicate.

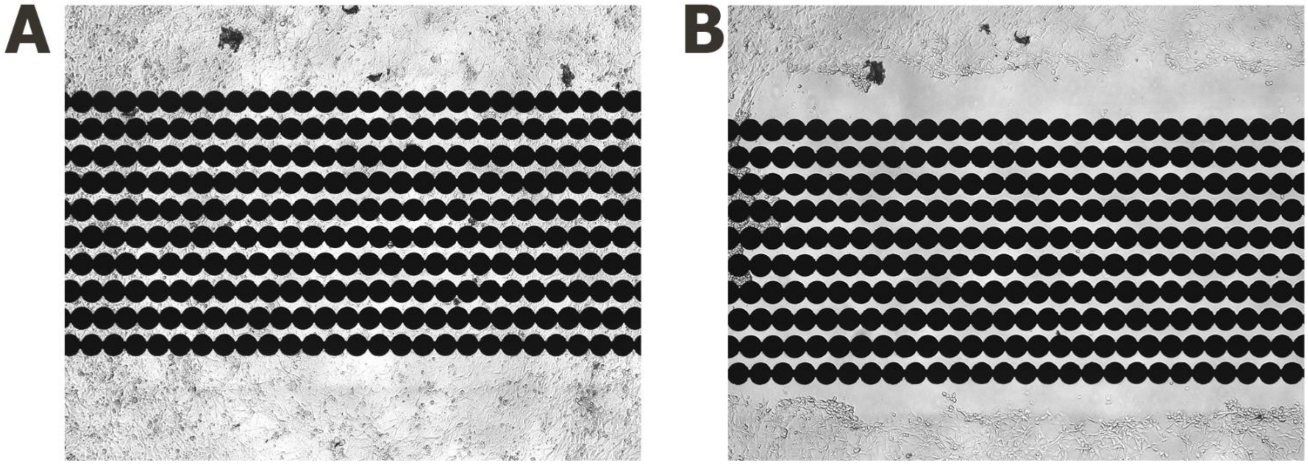

Sfigure 4. Brightfield images of HUVEC on a E-wound plate just before scratch (A) and directly after scratch induction with Accuwound 96 tool (B). Gold-electrodes are shown as black-dots that are not covered by cells anymore after the scratch.

2 Synthesis route of T26A:

T26A was synthesized according to procedures adapted from previously reported literature (**Scheme S1**)(Min et al., 2007; Schuster et al., 2015).

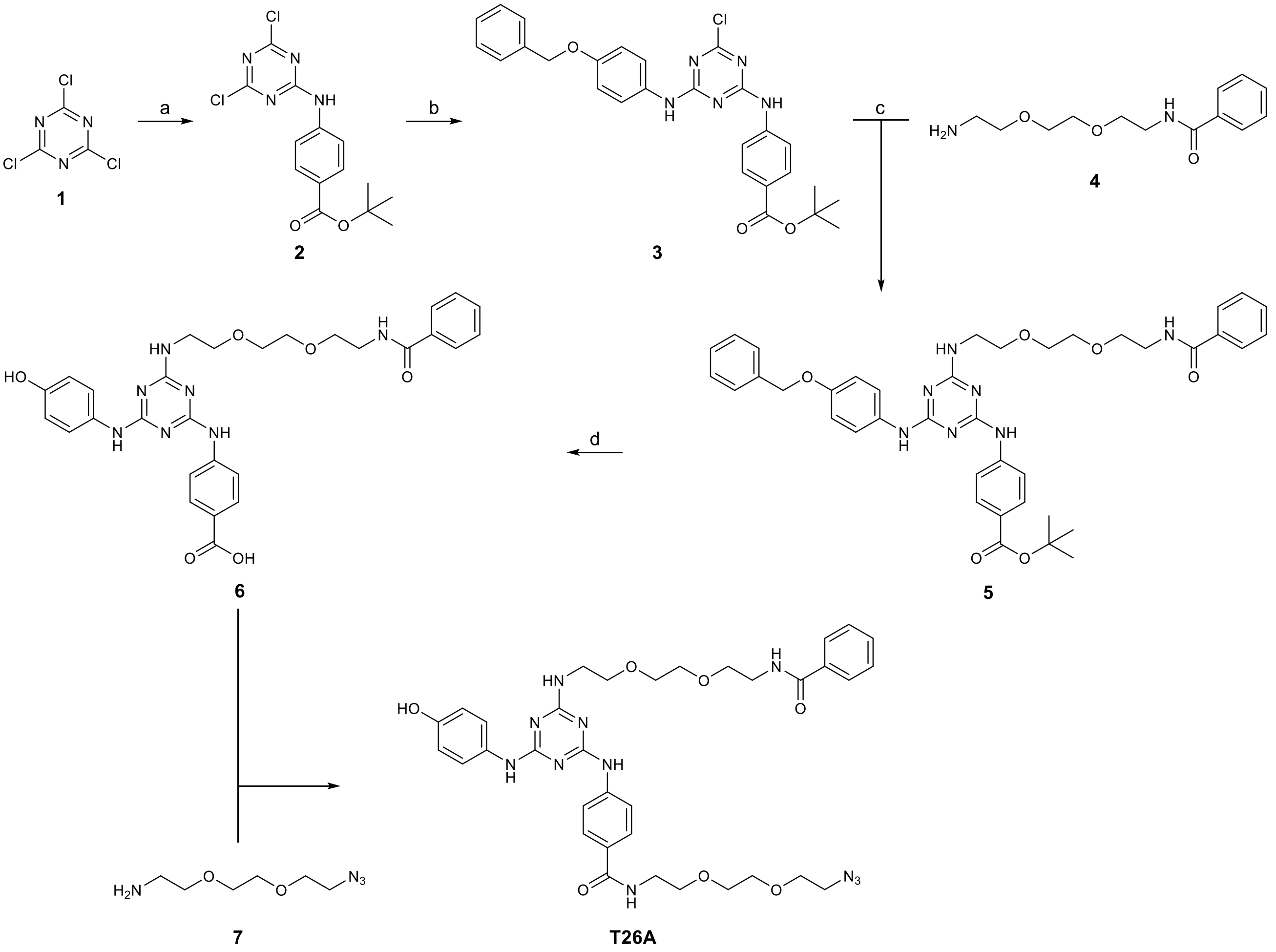

Scheme S1. Synthesis of prostaglandin transporter inhibitor **T26A**. Reagents and conditions: a) *Tert*-butyl-4-aminobenzoate, DIPEA, THF, 0 °C, 1 h (77%). b) 4-(benzyloxy)aniline hydrochloride, DIPEA, THF, 50 °C, 22 h (65%). c) DIPEA, THF, 65 °C, 16 h (42%). d) TFA, DMS, 50 °C, 23 h (56%). e) EDC HCl, HOBt, DIPEA, DMF, rt, 16 h (82%).

***Tert*-butyl 4-((4,6-dichloro-1,3,5-triazin-2-yl)amino)benzoate (2)**.

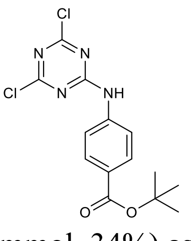

A solution 2,4,6-trichloro-1,3,5-triazine **1** (2.72 g, 14.8 mmol) and DIPEA (2.49 ml, 14.2 mmol) in anhydrous THF (30 ml) was cooled to 0 °C before dropwise adding a solution of *tert*-butyl-4-aminobenzoate (2.50 g, 12.9 mmol) in anhydrous THF (10 ml) over 15 min. The reaction mixture was stirred for 1 h under nitrogen atmosphere and was diluted with H_2O and EtOAc, resulting in a precipitate that was isolated by filtration and dried under reduced pressure to yield title compound **2** (1.5 g, 4.39 mmol, 34%) as a white solid. The water fraction was then extracted with EtOAc (3x). The combined organic fractions were washed with brine, dried over $MgSO_4$, filtered and concentrated under reduced pressure. The residue was triturated with EtOAc (30 ml) and dried under reduced pressure, to yield additional title compound **2**(1.91 g, 5.59 mmol, 33%) as a white solid.

^{1}H NMR (400 MHz, DMSO): δ 11.42 (s, 1H), 7.92 (d, J = 8.8 Hz, 2H), 7.75 (d, J = 8.8 Hz, 2H), 1.54 (s, 9H). **LC-MS:** R_t = 12.1 min (10-90% MeCN/H_2O, formic acid); found $[M+H]^+$ = 341.1 and $[M+H+MeCN]^+$ = 382.1.

***Tert*-butyl 4-((4-((4-(benzyloxy)phenyl)amino)-6-chloro-1,3,5-triazin-2-yl)amino)benzoate (3).**

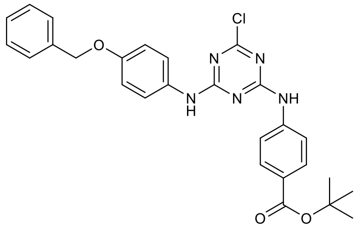

To a solution of **2** (3.41 g, 9.98 mmol) and 4-(benzyloxy)aniline hydrochloride (2.35 g, 9.98 mmol) in anhydrous THF (60 ml) was added DIPEA (5.23 ml, 29.9 mmol). The reaction mixture was stirred under nitrogen atmosphere at 50 °C for 6 h. Additional 4-(benzyloxy)aniline hydrochloride (0.24 g, 1.0 mmol) was added and the reaction mixture was stirred overnight at 50 °C. The suspension was filtered and the filtrate was concentrated under reduced pressure. Recrystallization from EtOAc/petroleum ether (4:1) provided title compound **3** (4.03 g, 6.50 mmol, 65% corrected for DIPEA HCl contamination) as a white solid.

^{1}H NMR (400 MHz, DMSO): δ 10.49 (s, 1H), 10.16 (d, J = 57.1 Hz, 1H), 9.23 (s, 1H), 8.00 – 7.70 (m, 5H), 7.56 – 7.28 (m, 7H), 7.03 (d, J = 8.4 Hz, 2H), 5.12 (s, 1H), 1.53 (d, J = 3.5 Hz, 9H). **LC-MS:** R_t = 13.4 min (10-90% MeCN/H_2O, formic acid); found $[M+H]^+$ = 504.2.

***Tert*-butyl-4-((4-((2-(2-(2-benzamidoethoxy)ethoxy)ethyl)amino)-6-((4-(benzyloxy)phenyl)amino)-1,3,5-triazin-2-yl)amino)benzoate (5).**

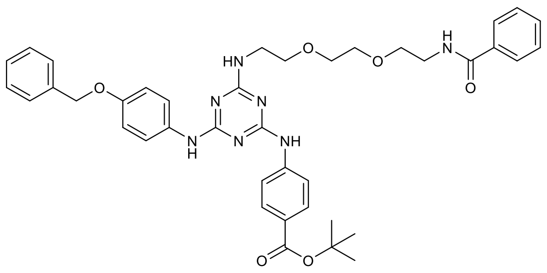

To a solution of **3** (1.96 g, 3.90 mmol) and **4** (Tang and Fang, 2008) (1.18 g, 4.68 mmol) in anhydrous THF (60 ml) was added DIPEA (2.0 ml, 11.7 mmol) before heating to 65°C. The reaction mixture was stirred overnight and the resulting suspension was filtered. The filtrate was concentrated under reduced pressure and the residue was subjected to silica gel column chromatography (2% MeOH/DCM) to yield title compound **5** (1.19 g, 1.65 mmol, 42%) as a white solid.

^{1}H NMR (400 MHz, $CDCl_3$): δ 7.94 – 7.84 (m, 2H), 7.76 (d, J = 7.0 Hz, 2H), 7.67 – 7.50 (m, 2H), 7.47 – 7.27 (m, 10H), 7.19 – 7.12 (m, 2H), 6.99 – 6.80 (m, 3H), 6.12 – 5.92 (m, 1H), 5.05 (s, 2H), 3.72 – 3.53 (m, 12H), 1.58 (s, 9H). **LC-MS:** R_t = 12.1 min (10-90% MeCN/H_2O, formic acid); found $[M+H]^+$ = 720.3.

4-((4-((2-(2-(2-Benzamidoethoxy)ethoxy)ethyl)amino)-6-((4-hydroxyphenyl)amino)-1,3,5-triazin-2-yl)amino)benzoic acid (6).

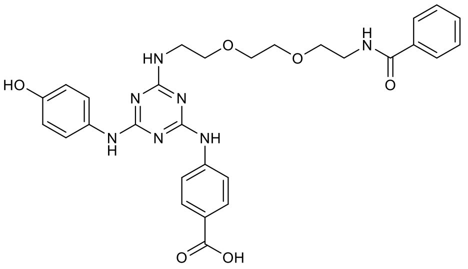

To a solution of **5** (1.19 g, 1.65 mmol) was added TFA (5.09 ml, 66.1 mmol) and dimethylsulfide (0.61 ml, 8.3 mmol). The reaction mixture was stirred at 50 °C for 23 h before diluting with H_2O. The water fraction was extracted with EtOAc (3x) and the combined organic fractions were dried over $MgSO_4$, filtered and concentrated under reduced pressure. The residue was subjected to silica gel column chromatography (10% MeOH/DCM) to yield the TFA salt of title compound **6** (842 mg, 0.920 mmol, 56%) as a white solid.

^{1}H NMR (400 MHz, DMSO): δ 9.97 (bs, 1H), 9.76 – 9.49 (m, 1H), 9.34 (bs, 1H), 8.53 (d, J = 6.1 Hz, 1H), 7.92 – 7.72 (m, 6H), 7.56 – 7.34 (m, 5H), 6.74 (dd, J = 16.3, 8.3 Hz, 2H), 3.56 (s, 8H), 3.54 – 3.37 (m,4H). **LC-MS:** R_t = 7.8 min (10-90% MeCN/H_2O, formic acid); found $[M+H]^+$ = 574.3.

4-((4-((2-(2-(2-Benzamidoethoxy)ethoxy)ethyl)amino)-6-((4-hydroxyphenyl)amino)-1,3,5-triazin-2-yl)amino)benzoic acid (T26A).

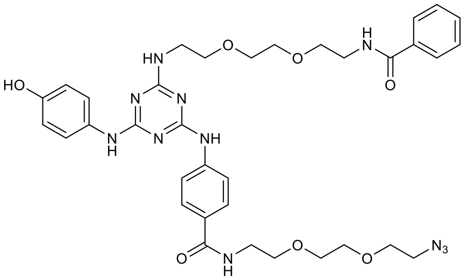

To a mixture of TFA salt **6** (0.84 g, 0.92 mmol) and **7** (Friscourt et al., 2012) (0.18 g, 1.0 mmol) in DMF (4 ml) were added EDC HCl (0.21 g, 1.1 mmol), HOBt (0.17 g, 1.1 mmol) and DIPEA (0.64 ml, 3.7 mmol). The reaction mixture was stirred at rt for 16 h under nitrogen atmosphere before diluting with H_2O. The water fraction was extracted with EtOAc (3x). The combined organic fractions were washed with brine (3x), dried over $MgSO_4$, filtered and concentrated under reduced pressure. The residue was subjected to silica gel column chromatography (5% MeOH/DCM) to yield title compound **T26A** (551 mg, 0.755 mmol, 82%) as a white solid. **^{1}H NMR** (400 MHz, MeOD): δ 7.82 – 7.69 (m, 6H), 7.48 – 7.28 (m, 5H), 6.75 (d, J = 8.5 Hz, 2H), 3.66 – 3.59 (m, 16H), 3.57 – 3.50 (m, 6H), 3.33 – 3.27 (m, 2H). **LC-MS:** R_t = 8.3 min (10-90% MeCN/H_2O, formic acid); found $[M+H]^+$ = 730.3.

References:

Friscourt, F., Fahrni, C. J., and Boons, G.-J. (2012). A Fluorogenic Probe for the Catalyst-Free Detection of Azide-Tagged Molecules. *J. Am. Chem. Soc.* 134, 18809–18815. doi: 10.1021/ja309000s.

Min, J., Kyung Kim, Y., Cipriani, P. G., Kang, M., Khersonsky, S. M., Walsh, D. P., et al. (2007). Forward chemical genetic approach identifies new role for GAPDH in insulin signaling. *Nat. Chem. Biol.* 3, 55–59. doi: 10.1038/nchembio833.

Schuster, V. L., Chi, Y., Wasmuth, A. S., Pottorf, R. S., and Olson, G. L. (2015). Prostaglandin Transporter Inhibitors and Uses Thereof. US8952150B2.

Tang, W., and Fang, S. (2008). Mono-acylation of symmetric diamines in the presence of water. *Tetrahedron Lett.* 49, 6003–6006. doi: https://doi.org/10.1016/j.tetlet.2008.07.174.
